# Editorial

**Published:** 1871-10

**Authors:** 


### EDITORIAL.

EXPLANATION.—The present number of the EXAMINER was just ready for delivery, but had not been sent out of the printing office, which was located in the North Division of the city, when the great fire occurred. It was consequently destroyed so completely that not even a scrap of the original manuscript was left. Some of the articles were soon reproduced and others substituted with a view to a speedy re-publication. But the fire had destroyed every press in the city in which book and medical journal work could be printed. We have waited patiently five or six weeks in the hope that our old printers would be ready to resume their work, but have at last been obliged to send to Ann Arbor for the printing of the present number. The November and December numbers will follow in quick succession, and by the first of January we expect to be on time with the first number of the new volume.

The subscription list and all the books belonging to the EXAMINER were preserved. We hope old subscribers who are in arrears will immediately pay up, and new ones send in their names for the next volume.

---

MEDICAL COLLEGES IN CHICAGO.—The Chicago Medical College, Medical Department of the Northwestern University, is located in the south part of the city, in the same block with the Mercy Hospital, and was entirely out of the reach of the fire; and as the students of this College were boarding in the same section of the city, they also escaped all injury or loss. Although Professors Isham and Byford suffered severely, losing both their residences and offices, and some of the other members of the faculty suffered minor losses, yet the regular lectures in the College were suspended only one day, and even on that day while the fire was raging with all its fury, Professor E. Andrews attended his clerical hour in the hospital and performed several operations in the presence of a large part of the class. The hour for these operations had been previously fixed, and their performance, together with the resumption of the full course of lectures the following morning, was not from any want of interest in the results of the fire or in the welfare of the community, but from the conviction that one of the most efficient modes of preventing demoralization and discouragment, when some great calamity has overtaken a community, is, for every man who has anything to do and a place left to do it in, to continue dilligently at his work, and employ as many others in the same way as possible. The class in attendance is fully equal to that of any previous year.

The Rush Medical College, being located in the North Division of the city was entirely consumed with all its contents. Several members of the faculty suffered very severe losses, and many of the students boarding in the vicinity also lost their books and clothing. Of course their lectures were suddenly suspended. On the next day after the fire their faculty were officially notified that all their matriculated students would be permitted to attend the lectures of the Chicago Medical College free of charge, until the faculty could procure new rooms in which to resume their lectures. And in case they did not deem it advisable to resume the present term, all their students who had actually paid their lecture fees were offered the privlege of completing their course in the Chicago Medical College without further charge.

In about two weeks the Faculty effected an arrangement by which they resumed their regular course of instruction, in the amphitheatre of the County Hospital. A large part of their students returned, and so far as we know, their work is progressing satisfactorily. In the present number of the EXAMINER will be found an appeal from the Visitors of that school, to its Alumni throughout the country, for aid to rebuild the College. We hope it will meet with a liberal response.

The Woman's Hospital Medical College was also burned out. Occupying temporary rooms and a recently organized institution, it had not much property to lose, and soon resumed its course of instruction in temporary quarters in the West Division of the city,

RELIEF FOR MEDICAL MEN—CHICAGO, October 19, 1871. —At a meeting of Physicians held on the 17th inst., at No. 797 Wabash avenue, of which Dr. N. S. Davis was made Chair man, and Dr. E. Andrews, Secretary; the announcement having been made that communications had been received from prominent Physicians of other cities, to the effect that contributions, for the relief of the suffering members of the profession here, are now awaiting the order of responsible parties to receive and disburse them. Drs. Moses Gunn, E. Andrews and A. Fisher having been appointed a committee to recommend suitable persons for a permanent Relief Committee of five, nominated the following gentlemen: Drs. N. S. Davis, DeLaskie Miller, Ernst Schmidt, T. D. Fitch and Walter Hay, which nominations being unanimously approved, the following resolutions were adopted:

*Resolved*, That the Committee just chosen is hereby authorized to receive all donations for the relief of the respectable Physicians, who are sufferers by the late fire, distribute the same at their discretion, and render a strict account, with vouchers, to any future meeting, which may be called by the Chairman, to consider the same.

*Resolved*, That this meeting tender the cordial and heartfelt thanks of the profession of this city to their brethren in other and distant cities, for the prompt and liberal offers of assistance to the many among us who have lost, by the late terrible fire, not only their homes, clothes, books and instruments but their practice, and pledge a just use of whatever is given.

Contributions may be forwarded at once by express, or draft on New York, to Walter Hay, M. D., Secretary Medical Relief Committee, No. 384 Michigan Avenue.

Donations from publishing houses, instrument makers and physicians, of books, instruments, or apparatus, will be gratefully received; as many of our professional brethren have saved only their lives.

DE LASKIE MILLER, M. D.,
No. 518 Wabash Avenue, Chairman.
N. S. DAVIS, M. D.,
No. 797 Wabash Avenue, Treasurer.
ERNST SCHMIDT, M. D.,
No. 387 State Street.
T. D. FITCH, M. D.,
No. 296 West Monroe Street.
WALTER HAY, M. D.,
No. 384 Michigan Avenue, Secretary.

} Committee.

Up to the date of this writing, November 18th, the above Committee had received $6,788.00, chiefly from New York, Brooklyn and Cincinnati; all but $1,000 of which has been distributed among some seventy-five members of the profession who had suffered severe losses by the fire. The sums given to individuals have varied from $30 to $135. In addition to this, the profession in St. Louis promptly contributed $900, which they sent in the hands of a committee of their own number, which committee apportioned it in sums of $50 each to eighteen of our physicians who had suffered most severely and were in most urgent need of aid. As near as we can learn, there were about one hundred regular physicians in good standing who lost almost everything in the shape of books, instruments and office furniture, and about half of them their residences and all the available property they possessed. Some of them had been doing laborious professional work for twenty and thirty years. Yet all are in good cheer, and more anxious to alleviate human suffering then to replenish their own pockets. What they feel most keenly the need of is books and instruments. Good, well assorted pocket-cases of surgical instruments and standard works on the practical departments of medicine would be highly acceptable to the afflicted part of the profession.

---

Chicago, Nov. 1, 1871.—To Whom it May Concern.—This is to certify, that the resident Alumni of Rush Medical College, of the city of Chicago, at a meeting held at the house of E. Ingals, M. D., on the eve of October 17, 1871, appointed the following Alumni an Executive Committee, to draft and present an appeal to the Alumni and friends of the College, for aid to rebuild and refurnish the College Building, viz: T. D. Fitch, M. D., Chairman; H. A. Johnson, M. D., V. L. Hurlbut, M. D , C. T. Parkes, M. D., Ben C. Miller, M. D., and F. A. Emmons, M. D.

E. Ingals, M. D., Chairman.
Curtis T. Fenn, M. D., Secretary.

An Appeal to the Alumni and Friends of the Rush Medical College, Recently Destroyed by Fire, for Aid to Assist in its Rebuilding.—This College is among the oldest institutions of learning in the northwest, having been in operation since 1843, at which time the region now tributary to Chicago was but sparsely populated, and had little wealth. During this time it has supplied a pressing need of this new country. It has educated a large number of young men, who are scattered through our whole country, worthily filling places of great usefulness and responsibility; and for this, both themselves and the public are indebted, in a great measure, to the school in which they received their instruction. A large proportion of its students have been possessed of little, save youth, hope, intelligence, and determination. Many of these, having been generously aided by the College, have taken rank among the most substantial members of the profession. The Faculty at all times, since its organization, has been moved by an earnest desire to promote the best interests of the profession and the College. For this its members have labored faithfully and earnestly; they have met the pecuniary burden of the school from its first foundation, and four years since they erected from their own resources, at an expense of $70,000, the most ample and best appointed college building on this continent, and filled it with every necessary appliance for successful teaching, and the influence and usefulness of the school has steadily increased from year to year. But in a day, the college building, with all its contents, was swept away, along with a large part of the city, in which it stood a peer among many other noble institutions of learning. The pecuniary loss of the Faculty, in the destruction of the college, is light when weighed against others they have sustained. A number have lost nearly everything, and all have suffered much. The college must be rebuilt. Its past history, its future promise for good, demand no less. Under the circumstances, it is unreasonable to expect the faculty to do this unaided. The college is now in a condition to justify an appeal to its Alumni, and to society, for some return for the favors it has conferred upon both. There is, perhaps, no field of benevolence, that offers a richer return than to provide adequate and easy opportunities for instruction to those who desire to become learned in the best means for assuaging pain and healing the sick.

All donations may be remitted to Charles T. Parkes, M. D., 462 Elston avenue, Chicago, who has been elected treasurer for the fund. They will be thankfully acknowledged, and faithfully devoted to the rebuilding of the college.

---

MEETING AND ORGANIZATION OF THE COMMITTEE.—At an appointed meeting of the Executive Committee, held at Cook County Hospital, October 26th, 1871, all the members being present, Dr. T. D. Fitch, in the chair, the committee organized by the election of the following officers: F. A. Emmons, M. D., Secretary; Ben C. Miller, M. D., Assistant Secretary; C. T. Parkes, M. D., Treasurer, who was required to give good and satisfactory bonds in the sum of thirty thousand ($30,000) dollars, for the faithful performance of his trust, which bond has been furnished and duly accepted.

T. D. FITCH, M. D., Chairman.
F. A. EMMONS, M. D., Secretary.

---

THE TRUSTEES OF RUSH MEDICAL COLLEGE TO ITS ALUMNI, GREETING.—The last terrible conflagration which devastated so large and fair a portion of Chicago, swept out of existence nearly all of the material part of your Alma Mater. Rush Medical College exists to-day only in its legal organization, the lot on which the College building stood, the energy of its Trustees and Faculty, and the love and fidelity of its Alumni.

The College edifice, so recently and expensively erected, the chemical and physiological laboratories, the museum, and all the appliances of teaching, are gone, and a sad material ruin replaces them.

The Trustees are, however, cheered and encouraged by the expressions of sympathy and offers of pecuniary assistance which have come to them from many of the Alumni, in different parts of the country. The Alumni in Chicago have appointed a committee, to appeal to their brethren, in behalf of their Alma Mater. This appeal the Trustees most heartily approve and endorse; and while all sums which may be offered will be most thankfully received, they are confident that fortune has smiled upon very many of the sons of "Old Rush," and that among these favored ones there are generous hearts, which will prompt to munificent donations. To such they make the following offer:

For every donation of five hundred dollars the Trustees will establish a perpetual free scholarship, which shall bear the name of the donor, and which shall be conspicuously emblazoned on the wall of the lecture room. A certificate of this scholarship, engrossed on parchment, will be issued to the donor; which certificate shall secure to the bearer, free tuition, and when found qualified, free graduation. This certificate shall be perpetual in its operation; and thus the donor will have endowed for one student each year a Free Medical College.

WM. B. OGDEN, Chairman.
GRANT GOODRICH, Secretary.

---

EFFECT OF CHLORAL HYDRATE ON THE BLOOD.—The London Microscopic Journal for August, 1871, contains an account of a number of experiments, made by Thos. S. Ralph. M.R., C.S., with a view of determining the chemical effects of Chloral Hydrate, Chloroform, Prussic Acid, and other agents, upon the blood.

The writer says: "Hydrate of chloral is a remarkable chemical substance, producing a singular effect on the blood when applied directly to it. A small portion being placed on a glass slide and slightly moistened, and then fluid blood added about one-third of the corpuscles appear to corrugate their solid contents, which then takes colour from magenta.

"In the following October I had occasion to administer chloral in small doses, with a view of relieving pain. This enabled me to examine the condition of the blood. The blood drawn two or three hours after its exhibition, presented a remarkable appearance. In several parts of the field of the microscope, besides garnet-coloured amorphous particles, a number of red-coloured globules (double the diameter of white corpuscles, and many smaller) were seen; some of them were dark red. This was experiment the first.

Experiment 2—Hydrate of Chloral was exhibited by the stomach to a rabbit; within an hour red masses were seen in the blood, also the presence of starchy bodies was noticed.

Experiment 3—A frog was subcutaneously injected with chloral hydrate, with the same results.

Experiment 4—A frog was immersed in a four-grain solution of the hydrate for some hours, when it was found hypnotic. Blood nuclei of corpuscles appeared greenish, red particles also seen.

Experiments 7 and 8—Two rats were killed, one by chloroform, the other by hydrate chloral, injected subcutaneously. In the latter the blood exhibited ruby-red particles; a few bluish; also starchy bodies in abundance. The urine also showed the same. The chloroformed rat presented an abundance of starchy bodies, and some blue-coloured particles; blood from lungs—plasma reddish; few starchy bodies; some blue particles; scarcely any reddish."

After detailing, in this manner, a large number of similar experiments the writer sums up his conclusions as follows:

" Hydrate of chloral administered by the stomach or subcutaneously injected, gives rise to the production of bright red or dark red particles, masses, or globules in the blood. Starchy bodies are also met with accompanying these changes. The urine also exhibits these bodies. The same result follows when vapor of chloral hydrate is applied to fresh drawn blood. Ammonia, administered by the lungs or subcutaneously during the action of chloral on the animal economy appears to heighten these effects.

Formic acid added to fresh blood also causes the production of dark red globules and particles.

Lactic acid conjoined with prussic acid does the same.

Prussic acid and ammonia conjoined yield the same results.

The action of hydrate chloral, while decomposing under ammonia or a salt of iron, presents changes which to my mind are identical. All these results I refer to the action of formyl or ammonia-formate on the iron of the blood. The decomposition of lactic acid with prussic acid can supply chemically the elements necessary for the production of formyl or formate of ammonia; as also prussic acid and ammonia."

Another remarkable occurrence is mentioned which receives its solution from the forementioned experiments. "Some blood was accidentally examined after wine (Reishing) had been taken; this was with a view of exhibiting the action of prussic acid on the iron in the blood; but it was noticed little or no reaction could be found after its application; but a good many red-coloured globules and particles were seen just as if chloral had been taken. In consequence of this, a small drop of urine was added to a little blood fresh drawn; the changes seen under the microscope were most remarkable. Abundance of globules of a dark-red or brown colour made their appearance, as also red amorphous masses or particles. Gas also was given off in the neighborhood of the globules. Some of these bubbles contained a bluish fluid; the nuclei of the white corpuscles were bluish.

The experience I have already gained in carrying out these experiments leads me to see that the condition of the blood recognizably varies from day to day, from the effects of food, etc.; for the varying degrees of success which have attended a number of experiments performed with the same chemical agent, on the same individual, point to the great probability of the variable condition of the blood, when that individual has been the subject of variety in diet, or degrees of fatigue of mind or body."

In concluding he says, "another consideration which presents itself to my mind is, that just as we now test the condition of the urine in order to ascertain what is being eliminated from the body of a patient, so will the physician find it useful occasionally to test, by means of reagents, the condition of the blood of his patient, in order to verify the character of some obscure symptoms. Even at this period of my experience, I have reason to believe it is possible by means of agents previously administered, to prolong the hypnotic action of chloral, or to prevent or modify it in a great degree. Thus, I believe, I have at least been able to give demonstration to the theory of Leibrich, who, by his chemical knowledge, has enabled the medical practitioner to employ a remarkable agent, one which promises to be a sister companion to chloroform in alleviating the ills to which flesh is heir. I hope I may be fortunate enough to arouse the attention of my professional brethren to the investigation of the chemical action of remedies on the blood, and thereby, perhaps, lead on to a more rational and satisfactory mode of treating some diseases, which in time to come, I believe will be attacked through the blood itself."

---

### PUBLICATIONS RECEIVED.

---

Plastics and Orthopedics: A Report re-published from the Transactions of the Illinois State Medical Society for 1871. By David Prince, M. D.

The report on Orthopedics made to the Illinois State Medical Society by Dr. Prince, in 1864, and his report on Plastics, made in 1867, were attempts to make the knowledge of these important branches of surgery more easily accessible to the profession. The present report is a continuation of this effort, such additions having been made as to include all the recent advances.

---

Transactions of the Indiana State Medical Society. Twenty-first Annual Session. 1871.

This is a handsome volume of 250 pages, containing a number of valuable and interesting reports. A finely executed likeness of the late John S. Bobb, M. D., with a short biographical sketch by G. W. Mears, M. D., also adds much to the value of the volume. We shall take occasion to give a more extended review of some of the reports in a future number.

